# Reference Values of Impulse Oscillometric Lung Function Indices in Adults of Advanced Age

**DOI:** 10.1371/journal.pone.0063366

**Published:** 2013-05-15

**Authors:** Holger Schulz, Claudia Flexeder, Jürgen Behr, Margit Heier, Rolf Holle, Rudolf M. Huber, Rudolf A. Jörres, Dennis Nowak, Annette Peters, H.-Erich Wichmann, Joachim Heinrich, Stefan Karrasch, the KORA Study Group

**Affiliations:** 1 Institute of Epidemiology I, Helmholtz Zentrum München, Munich, Germany; 2 Department of Internal Medicine V, Comprehensive Pneumology Center Munich, Ludwig-Maximilians-University, Munich, Germany; 3 Institute of Epidemiology II, Helmholtz Zentrum München, Munich, Germany; 4 Institute of Health Economics and Health Care Management, Helmholtz Zentrum München, Munich, Germany; 5 Division of Respiratory Medicine and Thoracic Oncology, Department of Medicine, Innenstadt, Ludwig-Maximilians-University, Munich, Germany; 6 Institute and Outpatient Clinic for Occupational, Social and Environmental Medicine, Ludwig-Maximilians-University, Munich, Germany; 7 Institute of Medical Data Management, Biometrics and Epidemiology, Ludwig-Maximilians-University, Munich, Germany; 8 Klinikum Grosshadern, Ludwig-Maximilians-University, Munich, Germany; 9 Comprehensive Pneumology Center Munich (CPC-M), Member of the German Center for Lung Research, Munich, Germany; Pulmonary Research Institute at LungClinic Grosshansdorf, United States of America

## Abstract

**Background:**

Impulse oscillometry (IOS) is a non-demanding lung function test. Its diagnostic use may be particularly useful in patients of advanced age with physical or mental limitations unable to perform spirometry. Only few reference equations are available for Caucasians, none of them covering the old age. Here, we provide reference equations up to advanced age and compare them with currently available equations.

**Methods:**

IOS was performed in a population-based sample of 1990 subjects, aged 45–91 years, from KORA cohorts (Augsburg, Germany). From those, 397 never-smoking, lung healthy subjects with normal spirometry were identified and sex-specific quantile regression models with age, height and body weight as predictors for respiratory system impedance, resistance, reactance, and other parameters of IOS applied.

**Results:**

Women (n = 243) showed higher resistance values than men (n = 154), while reactance at low frequencies (up to 20 Hz) was lower (p<0.05). A significant age dependency was observed for the difference between resistance values at 5 Hz and 20 Hz (R5–R20), the integrated area of low-frequency reactance (AX), and resonant frequency (Fres) in both sexes whereas reactance at 5 Hz (X5) was age dependent only in females. In the healthy subjects (n = 397), mean differences between observed values and predictions for resistance (5 Hz and 20 Hz) and reactance (5 Hz) ranged between −1% and 5% when using the present model. In contrast, differences based on the currently applied equations (Vogel & Smidt 1994) ranged between −34% and 76%. Regarding our equations the indices were beyond the limits of normal in 8.1% to 18.6% of the entire KORA cohort (n = 1990), and in 0.7% to 9.4% with the currently applied equations.

**Conclusions:**

Our study provides up-to-date reference equations for IOS in Caucasians aged 45 to 85 years. We suggest the use of the present equations particularly in advanced age in order to detect airway dysfunction.

## Introduction

Forced oscillation techniques (FOT), in particular impulse oscillometry (IOS), have been introduced as complementary approach to conventional pulmonary function testing [1]–[3]. FOT determines mechanical characteristics of the respiratory system via the assessment of impedance, providing the real and the imaginary part, i.e. resistance and reactance, respectively, of impedance over a wide range of frequencies. It is an effort-independent and patient-friendly technique, requiring only minimal cooperation by the patient without special breathing maneuvers.

Therefore, FOT is well accepted by pediatricians for pulmonary function testing in young children who cannot perform spirometry properly [4]–[6], and its feasibility and clinical usefulness have been demonstrated [4]–[6]. In adults, its diagnostic value for obstructive lung disease in comparison to spirometry or body plethysmography is still controversial (e.g. [7]–[11]). Despite this it could be suspected that FOT is especially suited for lung function testing in elderly patients who cannot perform demanding maneuvers due to mental or physical impairments [11]. Pezzoli and colleagues [12] showed that almost 20% of patients aged between 65 and 94 years were unable to perform spirometry satisfying ATS/ERS requirements. Moreover, the time needed for spirometry was as long as 20 to 30 minutes which means a handicap for both clinical use and epidemiological studies. FOT may serve as an alternative in these patients in view of the fact that the population is getting older, lung diseases are becoming more frequent [13] and feasible diagnostic tools are needed.

Although total respiratory impedance is known to depend on age [14], only few reference studies exist in adults [2], [3], [9], [15]–[17], with only one covering the advanced age for IOS indices in Japanese adults [17]. Reduced lung elasticity and increased stiffness of the chest wall contribute to an impairment of lung function with age. Further characteristics are airspace dilatation and increased collapsibility of small airways [18], [19]. Correspondingly, forced vital capacity (FVC) and forced expiratory volume in 1 second (FEV_1_) decline while residual volume increases. In contrast, airways resistance as measured by body plethysmography is much less age-dependent than spirometric indices [20].

Based on this, the aim of the present study was to determine the age-dependence of IOS indices in healthy subjects of middle and advanced age and to establish up-to-date reference equations. For this we used two representative population samples from the South of Germany in which IOS measurements had been carried out in 1990 subjects covering an age range for reference equations from 45 to 85 years.

## Methods

### Ethics Statement

The KORA studies (Cooperative Health Research in the Augsburg Region, Germany) were approved by the Ethics Committee of the Bavarian Medical Association and written informed consent was obtained from the study participants.

### Subjects and Lung Function

Spirometry and IOS were determined in two cohorts of age 45 to 65 years (KORA-F4L, n = 1,050) and 65 to 91 years (KORA-AGE 1, n = 960) within a representative population cohort of the Augsburg region [21]–[23]. KORA F4L is the 3-year follow-up of the KORA F4 cohort, where spirometry has been carried out in 1,321 subjects between 2006 and 2008 [24]. Due to decease, relocation, or other reasons 1,293 subjects have been invited for the F4L follow-up in 2009. From those 1,050 subjects (81.2%) performed spirometry and IOS measurements in KORA-F4L. The entire KORA AGE 1 cohort is comprised of 5,991 subjects from which a randomly selected sample of 1079 participants has been invited for physical examination to the study centre [23]. Due to physical conditions, contraindications, or exhaustion 119 subjects had to be excluded from lung function so that 960 subjects (89%) were examined by spirometry and IOS. Both studies were approved by the Ethics Committee of the Bavarian Medical Association and informed consent was obtained from the study participants. Conditions for lung function measurements including the main examiners were the same in both cohorts with IOS measurements preceding spirometry. Standing height and weight were measured on the day of examination while subjects were wearing light clothes without shoes.

Standard spirometry was performed in line with ATS/ERS recommendations [25] in a sitting position using the Masterscope PC spirometer (Erich Jaeger/CareFusion, Germany) while subjects were wearing nose clips. The Lilly-type pneumotachograph was calibrated daily using a calibration syringe supplied and certified by the manufacturer. Additionally, daily self-testing of the examiners (biological control) was performed. Under the guidance of the experienced examiners at least 3 and at most 8 trials were recorded to obtain at least 2 acceptable and reproducible flow-volume curves. After completion of each test, the curves were visually inspected, maneuvers with artefacts excluded and results evaluated according to ATS/ERS recommendations [25]. Spirometric indices measured were maximal forced expiratory volume in 1 second (FEV_1_), maximal forced vital capacity (FVC), and the Tiffeneau index FEV_1_/FVC.

IOS measurements and their quality control were conducted in line with ERS Task Force recommendations [3] using the Masterscreen IOS (Erich Jaeger/Care Fusion, Germany). For quality control the Lilly-type pneumotachograph flow transducer (screen resistance of 36 Pa^.^s^.^L^−1^, flow accuracy ±1 mL; volume computer integrated, accuracy 1 mL) was calibrated daily using a calibration syringe supplied and certified by the manufacturer. Additionally, a reference impedance (0.2 kPa s L^−1^) provided by the manufacturer was used every two weeks to check the pressure transducer calibration and the overall spectral accuracy of the IOS set-up. Throughout the measurement period values for Rrs (0.2 kPa s L^−1^) and Xrs (0.0 kPa s L^−1^) spectra were frequency independent and variations smaller than the recommended 10% or 0.01 kPa s L^−1^. IOS measurements were performed in a sitting position. A nose clip was used and subjects were asked to support their cheeks with their palms and keep their lips sealed tightly around the mouth piece while breathing quietly. After adaptation to the setup, the subjects performed a minimum of 3 consecutive measurements of 30 s each with an impulse interval of 0.2 seconds, i.e. 5 impulses per second. During data acquisition time trends of flow, volume, and respiratory system impedance at 5 Hz (Zrs5) were monitored online by the examiner. After each test, respiratory system resistance (Rrs), reactance (Xrs) and the coherence (Co) between 5 Hz and 35 Hz as well as the volume dependence of Zrs5 were visually inspected and checked for artefacts, such as irregular breathing, hyperventilation, leakages or swallowing. Measurements with artefacts were discarded and, if possible, within the time schedule repeated.

As outcome measures we used respiratory system impedance at 5 Hz (Zrs), resistance (Rrs) and reactance (Xrs) between 5 Hz and 35 Hz in 5 Hz increments (R5–R35 and X5–X35, respectively), resonant frequency (Fres), integrated area of low-frequency reactance (AX) from zero line between X5 and resonant frequency, the absolute difference of R5 and R20 (R5–R20), and the relative difference ((R5–R20)/R20).

IOS was performed in 1990 subjects. From those, IOS measurements with coherence values below 0.6 between 5 Hz and 15 Hz or below 0.7 for frequencies >20 Hz were rejected, as well as measurements with atypical resting breathing patterns, i.e. tidal volume above 2.5 L for men and 2.0 L for women or a breathing frequency above 30 min^−1^ [3]. Although care was taken to avoid artefacts during measurements normal spirometry associated with extreme resistance and reactance values and abnormal flow [26] were suspicious for artefacts during the Zrs estimates. In case a measurement is considered artefactual, the guidelines recommend rejection of both, Rrs and Xrs [3]. Accordingly and to be on the safe side, those measurements were discarded from analysis (139 subjects). Further, within subject variability as assessed by the coefficient of variation of Zrs5 had to be smaller than 15%, with typical values below 10%. As a result of all quality criteria, 1811 subjects with valid IOS measurements were left.

From those 397 lung healthy subjects, aged 45 to 89 years, were identified after exclusion of subjects with spirometric values below the lower limit of normal [24], acute respiratory tract infections, a history of allergic rhinitis, asthma, or COPD (due to symptoms, medication (inhaled beta-agonists, steroids, and/or anticholinergic drugs) and/or diagnosed by a physician), and ever-smokers. These conditions were assessed by questionnaire or from the Pharmazentralnummer (PZN) of the medication taken. Questionnaires and information on medication (beta-blockers, diuretics, and antiarrhythmics) were used to assess the prevalence of cardiac diseases covering myocardial infarction, heart failure, coronary artery disease, and cardiac arrhythmias including atrial fibrillation.

### Statistical Analyses

Quantile regression models [27] were used to calculate sex specific reference equations for IOS indices. In contrast to linear regression, quantile regression requires no distributional assumption and therefore is more robust to outlying observations. Thus, it allows an adequate estimation of percentiles of the outcome [28].

In the present study, prediction equations were derived separately for the median as well as the 5th and 95th percentile, i.e. the lower and upper limit of normal (LLN and ULN, respectively) because most of the IOS indices exhibited no normal distribution (Shapiro-Wilk test). From the methodological point of view the 2.5^th^ and 97.5^th^ percentiles would be more appropriate for normalcy but conventionally the 5^th^ and 95^th^ percentiles have been used in respiratory medicine (e.g. [20], [29]). Age and height were included as independent variables in the model and the additional influence of weight was tested using the analysis of deviance. Beside linear models we also tested more complex regression models with quadratic terms of age, height and weight. It turned out, however, that the linear models fitted best according to the analysis of deviance.

The derived reference equations were compared to prediction equations for IOS indices proposed by Newbury et al. [15] and Vogel and Smidt [2]. Differences between predicted and observed values are presented as mean difference in percent of mean observed values and mean squared difference. Furthermore, the proportion of observed values above the predicted upper limit of normal (ULN) and below the lower limit of normal (LLN), respectively, were calculated. Bland Altman plots [30] illustrate the differences between predicted values based on the equations from the current study and those from other studies. Additionally, predicted values for selected resistance and reactance between 5 and 35 Hz for males and females at the age of 50 and 80 years were calculated using reference equations from the present study and other studies based on mean height and weight.

Differences between males and females for resistance and reactance values at different frequencies were tested using Wilcoxon rank sum test and p-values below 0.05 are used to indicate statistical significance. All analyses were performed using the statistical software R, version 2.15.1 (http://www.r-project.org) [31].

## Results

### Study Population

From the 1990 subjects studied 179 did not fulfil the quality criteria due to suspicion of mouth piece leakage, atypical resting breathing patterns, low coherence values, or large within subject variability leaving 1811 subjects. Due to the strict inclusion criteria 397 of those subjects were considered as lung healthy, with a sex ratio of 1.5 to 1 in favour of females (Table 1). FEV_1_, FVC, and the FEV_1_/FVC ratio showed an age-dependent decline between KORA F4L and AGE. Percent predicted values of both cohorts were within the normal range. Tidal volume (Vt) and respiratory rate (BF) reflected resting conditions but upper and lower limits indicated extremes that occur during lung function testing of large samples. Both sexes showed an equal age distribution throughout the age range of 45 to 85 years, with rarefication at older ages (Table 2). The aged subjects addressed in the study represented almost 60% of the population. The exclusion rates were relatively evenly distributed throughout ages but lower in females than in males mainly due to higher rates of never smokers in females.

**Table 1:** Descriptive data of the samples from KORA-F4L and KORA-AGE, median (5th, 95th percentile).

	KORA-F4L				KORA-AGE			
	Men		Women		Men		Women	
n	68		94		86		149	
Age (years)	54.00	(46.00, 63.00)	55.00	(46.00, 63.00)	73.50	(66.00, 84.75)	75.00	(66.00, 85.00)
Height (cm)	176.85	(167.34, 188.41)	163.35	(153.67, 172.24)	170.50	(163.00, 181.00)	158.00	(149.00, 167.60)
Weight (kg)	84.55	(67.02, 106.76)	68.95	(54.09, 91.05)	78.00	(68.25, 97.00)	68.00	(53.40, 87.60)
BMI (kg/m^2^)	27.26	(22.28, 33.86)	25.70	(20.60, 33.62)	26.62	(23.69, 33.07)	27.34	(21.56, 34.61)
Prevalence of cardiac diseases (n, %)	3	(4.4)	8	(8.5)	14	(16.3)	27	(18.1)
Tidal volume (L)	1.17	(0.64, 2.16)	0.89	(0.46, 1.57)	1.07	(0.44, 1.98)	0.80	(0.39, 1.44)
Breathing frequency (min^−1^)	12.98	(9.46, 19.92)	15.55	(8.96, 22.76)	14.68	(10.07, 21.23)	16.51	(10.08, 23.63)
FEV_1_ (L)	3.98	(3.21, 5.18)	2.92	(2.28, 3.73)	3.25	(2.42, 4.17)	2.22	(1.62, 2.87)
FVC (L)	5.13	(4.26, 6.56)	3.70	(2.80, 4.73)	4.23	(3.16, 5.36)	2.82	(2.17, 3.70)
FEV_1_/FVC	0.79	(0.74, 0.85)	0.79	(0.73, 0.86)	0.76	(0.69, 0.87)	0.78	(0.69, 0.87)
FEV_1_ (pp)	101.77	(83.60, 117.39)	101.22	(86.09, 122.10)	104.84	(85.98, 123.51)	103.43	(84.02, 125.59)
FVC (pp)	101.97	(84.69, 119.60)	100.66	(84.95, 123.06)	103.78	(82.30, 120.73)	100.41	(82.66, 125.75)
FEV_1_/FVC (pp)	98.69	(93.06, 105.20)	99.76	(92.24, 108.79)	99.94	(90.14, 113.90)	100.37	(89.90, 112.42)

Provided are median and 5th, 95th percentile in brackets, pp: percent predicted according to Karrasch et al. [24]. Cardiac diseases are covering myocardial infarction, heart failure, coronary artery disease, and cardiac arrhythmias including atrial fibrillation.

**Table 2:** Age distribution of the combined sample from KORA-F4L and KORA-AGE.

Age, years	Men			Women		
	included		excluded	included		excluded
45–54	36	(23.4)	247 (85.4)	43	(17.7)	255 (83.1)
55–64	32	(20.8)	249 (87.1)	51	(21.0)	293 (82.6)
65–74	45	(29.2)	207 (78.3)	69	(28.4)	209 (66.8)
75–84	36	(23.4)	227 (84.1)	70	(28.8)	215 (67.4)
≥85	5	(3.2)	44 (88.6)	10	(4.1)	45 (77.8)

Given are the absolute number and the percentage of subjects [n (%)] included and excluded due the selection criteria provided in methods.

### Frequency Dependency of Resistance and Reactance

At all frequencies women showed higher resistance values than men (p<0.05), while reactance at low frequencies, up to 20 Hz, was lower (p<0.05, Figure S1). Most of the IOS indices, particularly resistance indices, were not normally distributed.

### Age Dependency of IOS Indices

Individual values from all subjects as a function of age are provided in Figure S2. Moreover, for a ficticious subject showing median height and weight of the study population (phantom subject), lower and upper limits of normal (5th and 95th percentile) and the median were evaluated by our newly derived reference equations. Among the IOS indices, a significant age dependency was observed for the difference between R5 and R20, AX, Fres in both sexes and X5 in females.

### Reference Equations

**Figure 1:**
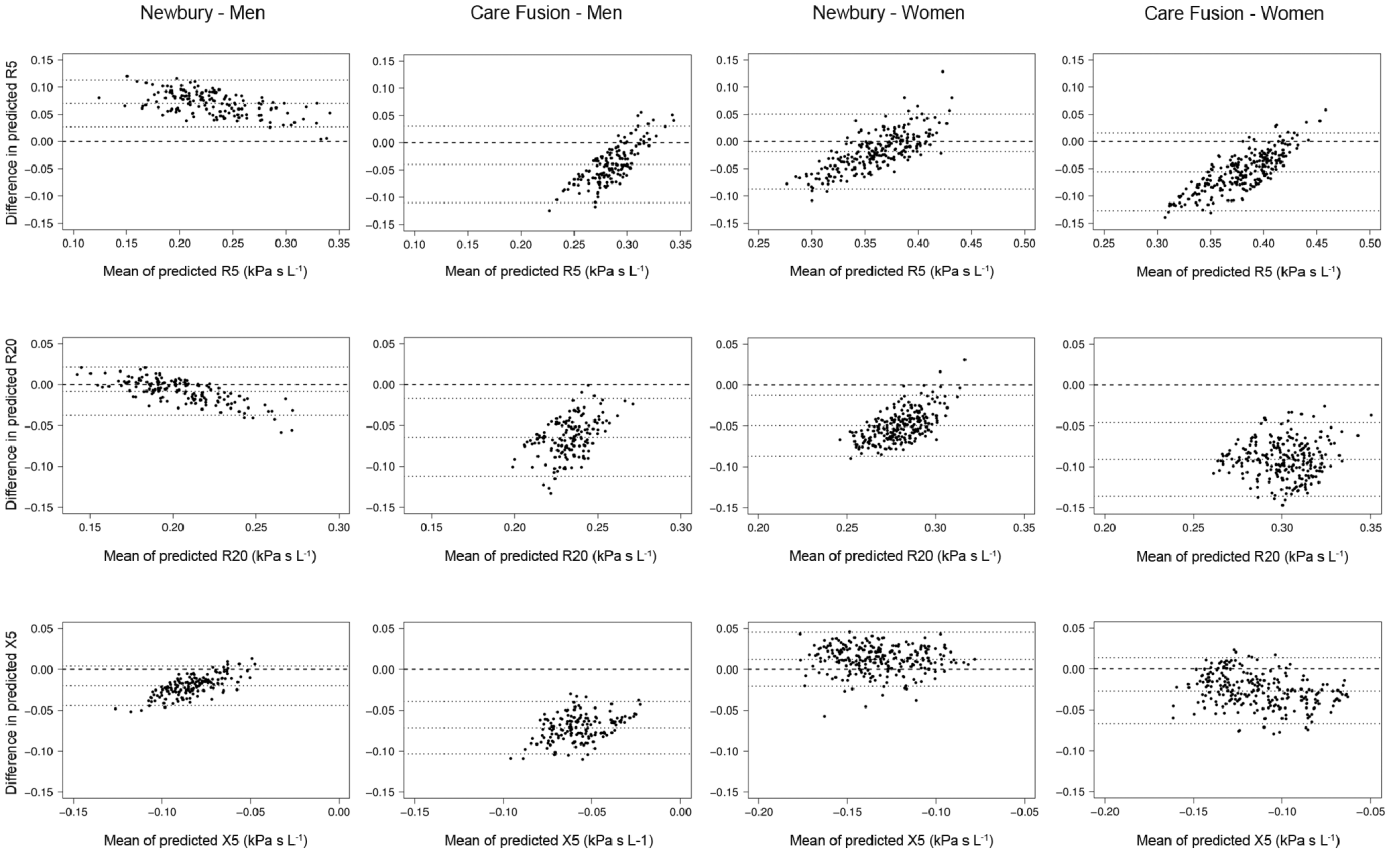
Bland-Altman plots for R5, R20, and X5. Vogel & Smidt 1994 (Care Fusion) [2], Newbury et al. 2008 [15].

**Figure 2:**
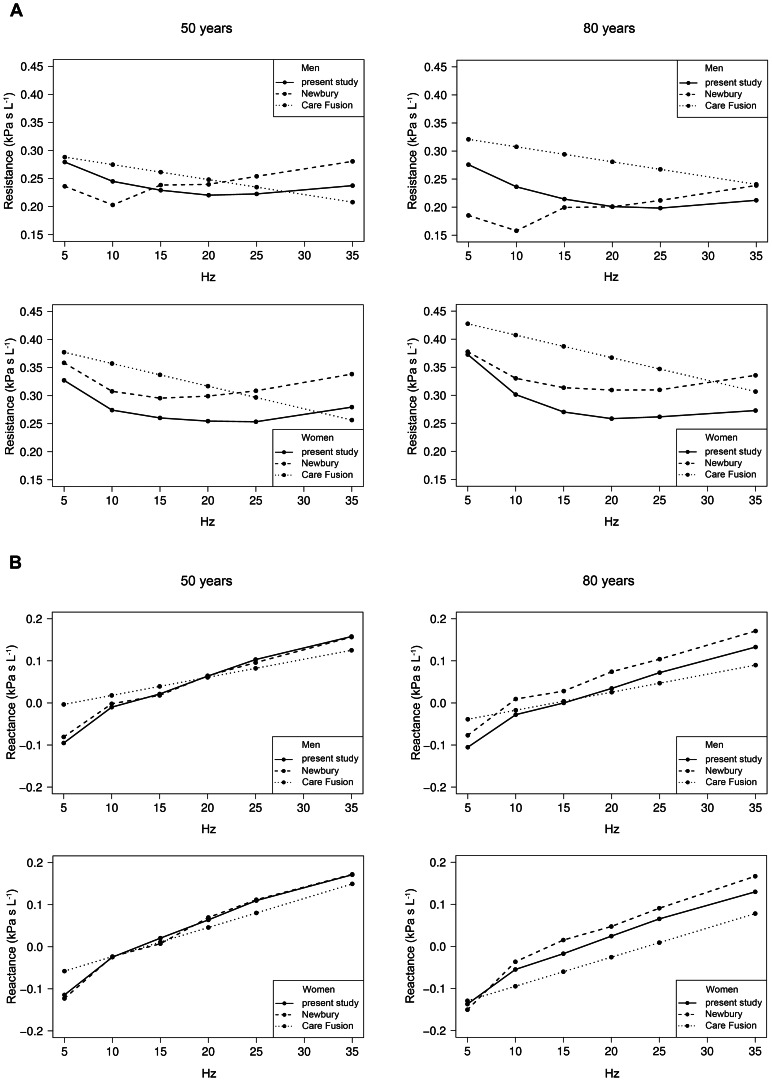
Comparison of values for resistance (A) and reactance (B) for 50 and 80 year old men and women. Calculations are based on equations from Newbury [15] and Vogel & Smidt 1994 (Care Fusion) [2] using median height and weight values from our study population.

For both sexes, predictive equations for the median, the 5th and 95th percentile of commonly applied IOS indices are provided in Table 3 (men) and Table 4 (women); the equations for other indices are given in the supplement, Table S1. Body weight more often contributed to the model statistically significantly in women than in men. Exclusion of subjects with cardiac diseases did not affect the reference equations significantly. This may be related to the fact that participants visiting the study center typically have early and less severe disease stages which may not affect lung function. Comparisons of the measured resistance and reactance data with the equations by Newbury et al. (n = 125, [15]), and Vogel & Smidt (n = 584, [2]) are illustrated in Figure 1. Regarding the Newbury equations, the Bland-Altman plots for R5, R20 and X5 showed obvious systematic differences for both sexes, except for R20 in men and X5 in women. Correspondingly, frequency-dependent resistance and reactance for 50 and 80 years old phantom subjects showed clear differences for R5, whereas those for X5 appeared to be smaller (Fig. 2).

**Table 3:** Reference equations of commonly applied IOS indices for men.

Equations				Coefficients of		
Percentile		Quantile value	Intercept	Age	Height	Weight
Z5 (kPa s L^−1^)	5%	0.18	1.2018022	−0.0025997	−0.0048804	
	50%	0.28	1.0719341	−0.0001490	−0.0059151	0.0029873
	95%	0.47	0.6514286	0.0035450	−0.0026455	
R5 (kPa s L^−1^)	5%	0.17	1.0685571	−0.0022403	−0.0043124	
	50%	0.26	0.9861137	−0.0001223	−0.0055278	0.0029891
	95%	0.44	0.6683472	0.0029051	−0.0026280	
R20 (kPa s L^−1^)	5%	0.13	0.6342369	−0.0019656	−0.0021069	
	50%	0.20	0.7722257	−0.0006446	−0.0037120	0.0013924
	95%	0.32	1.1824320	−0.0003590	−0.0048639	
R5–R20 (kPa s L^−1^)	5%	0.02	0.0358593	0.0003691	−0.0002018	
	50%	0.06	0.3140859	0.0004753	−0.0024277	0.0016455
	95%	0.13	0.0705078	0.0014497	−0.0002216	
(R5–R20)/R20*100 (%)	5%	11.57	37.8410856	0.2236991	−0.2295525	
	50%	29.92	31.9858256	0.4017027	−0.4662949	0.6370333
	95%	61.12	−2.6268375	0.4546831	0.1793720	
X5 (kPa s L^−1^)	5%	−0.15	−0.5568254	−0.0004762	0.0025397	
	50%	−0.09	−0.4670275	−0.0003344	0.0027755	−0.0010424
	95%	−0.04	−0.2058333	0.0006944	0.0006944	
AX (Hz kPa s L^−1^)	5%	0.07	0.0430162	0.0012195	−0.0002327	
	50%	0.26	1.7584772	0.0027451	−0.0132469	0.0077552
	95%	0.83	2.2955939	0.0161916	−0.0139157	
Fres (Hz)	5%	8.48	26.6835318	0.0582454	−0.1720116	0.1003766
	50%	13.35	24.9095582	0.1031097	−0.1874266	0.1675556
	95%	18.53	10.1428126	0.1772368	−0.0120106	

Equations are derived from lung healthy men aged 45 to 89 years. Quantile regression equations for median, 5th and 95th percentile with age in years, height in cm and weight in kg (e.g., Z5–50% = 1.0719341–0.0001490 age–0.0059151 height +0.0029873 weight).

**Table 4:** Reference equations of commonly applied IOS indices for women.

Equations				Coefficients of		
Percentile		Quantile value	Intercept	Age	Height	Weight
Z5 (kPa s L^−1^)	5%	0.23	0.6438520	−0.0002790	−0.0042285	0.0043481
	50%	0.37	0.9821420	0.0015343	−0.0059884	0.0035971
	95%	0.52	0.5504061	0.0012158	−0.0007407	
R5 (kPa s L^−1^)	5%	0.21	0.5012224	0.0002940	−0.0037866	0.0045199
	50%	0.34	0.7887960	0.0015118	−0.0046594	0.0029768
	95%	0.49	0.1926787	0.0004133	0.0016756	
R20 (kPa s L^−1^)	5%	0.16	0.5970057	−0.0006897	−0.0030934	0.0016623
	50%	0.26	0.4505645	0.0001251	−0.0020488	0.0017939
	95%	0.38	0.8177947	−0.0003549	−0.0025771	
R5–R20 (kPa s L^−1^)	5%	0.02	0.1121160	0.0000864	−0.0012551	0.0016042
	50%	0.09	0.2903621	0.0010200	−0.0023036	0.0013466
	95%	0.18	0.2123060	0.0009332	−0.0015425	0.0019627
(R5–R20)/R20*100 (%)	5%	8.12	24.5324087	0.1010746	−0.3020042	0.4036923
	50%	34.13	97.7280594	0.2997946	−0.7060959	0.4350743
	95%	69.92	154.3611632	0.1383460	−0.9307476	0.7594528
X5 (kPa s L^−1^)	5%	−0.22	−0.5446960	−0.0018068	0.0029323	
	50%	−0.12	−0.3313017	−0.0007541	0.0022090	−0.0014134
	95%	−0.07	−0.1831886	0.0003607	0.0005743	
AX (Hz kPa s L^−1^)	5%	0.14	1.0968758	−0.0001349	−0.0077805	0.0048793
	50%	0.50	1.7909627	0.0110077	−0.0168559	0.0104360
	95%	1.47	2.7124098	0.0173679	−0.0227368	0.0152978
Fres (Hz)	5%	9.10	27.7039789	0.0406373	−0.1748922	0.1125957
	50%	15.08	29.3243480	0.1446923	−0.1998960	0.1180629
	95%	22.31	22.5107342	0.1886975	−0.0871522	

Equations are derived from lung healthy women aged 45 to 89 years. Quantile regression equations for median, 5th and 95th percentile with age in years, height in cm and weight in kg (e.g., for women Z5–50% = 0.9821420+0.0015343 age–0.0059884 height +0.0035971 weight).

In our lung healthy study population mean differences between observed values and predictions were marked for R5 and X5 in men when comparing Newbury with our equations, 29.2% versus 3.9% and 21.3% vs. −0.3%, respectively (Table 5). In women distinct differences were observed for R20 (−17.8% vs. 1.4%). Obvious differences to Vogel & Smidt equations [2] were detected for R20 and X5 in both, men (−25.5% vs. 5.2% and 76.3% vs. −0.3%, respectively) and in women (−33.8% vs. 1.4% and 22.6% vs. 2.0%, respectively). The percentages of values above the ULN or below the LLN (5% expected by definition) also varied. In men much higher values were achieved for R5 (16.2% vs. 5.8%) and X5 (13.0% vs. 3.9%) when compared to Newbury et al. With respect to the Vogel & Smidt equations, almost no in women and only a low percentage in men (<1.3%) were beyond the limits of normal.

**Table 5:** Differences between observed values and predictions in lung healthy individuals.

Parameter	Study	Mean difference	Mean difference, %	mean squared difference	Observed values above ULN / below LLN, %
**Men (n = 154)**					
R5†	present study	0.0106	3.86	0.0054	5.84
	Newbury	0.0806	29.20	0.0125	16.23
	Care Fusion	-0.0293	-10.61	0.0072	1.30
R20†	present study	0.0111	5.23	0.0034	4.55
	Newbury	0.0031	1.48	0.0037	3.90
	Care Fusion	-0.0534	-25.24	0.0067	1.30
X5^‡^	present study	0.0003	-0.32	0.0009	3.90
	Newbury	-0.0199	21.35	0.0014	12.99
	Care Fusion	-0.0710	76.34	0.0061	0.65
**Women (n = 243)**					
R5†	present study	-0.0029	-0.83	0.0061	4.12
	Newbury	-0.0210	-6.04	0.0075	0.00
	Care Fusion	-0.0584	-16.77	0.0105	0.00
R20†	present study	0.0037	1.43	0.0037	5.35
	Newbury	-0.0460	-17.77	0.0060	0.00
	Care Fusion	-0.0875	-33.77	0.0117	0.00
X5^‡^	present study	-0.0026	2.01	0.0017	4.53
	Newbury	0.0098	-7.56	0.0021	1.23
	Care Fusion	-0.0292	22.61	0.0030	0.41

† above upper limit of normal (ULN), ^‡^ below lower limit of normal (LLN).

To further characterize differences in the discriminative power between the equations, comparisons between observed and predicted values and the number of subjects beyond the limits of normal were performed in the entire F4L and AGE population with acceptable IOS measurements (n = 1811, Table 6). Mean differences of percentages in men ranged between −17.7% and 81.4% and −24.7% and 38.6% in women. With respect to Newbury et al. the percentage of values above the ULN was higher for R5 in men (21.6% vs. 18.5%) and half as much for R20 (5.9% vs. 12.8%) compared to our equations. Substantial differences also occurred for X5 in men (24.9% vs. 15.1%). In women, lower values were seen for R5 (5.3% vs. 16.0%), for R20 (1.4% vs. 8.1%) as well as for X5 (8.3% vs. 18.6%). With regard to the Vogel and Smidt equations, values for R5, R20 as well as X5 were much lower in both sexes compared to our equations. Thus, the number of subjects with IOS values beyond the limits of normal depended substantially on the reference equations used.

**Table 6:** Differences between observed values and predictions for the KORA-F4L and KORA-AGE study population.

Parameter	Study	Mean difference	Mean difference, %	mean squared difference	observed values above ULN / below LLN, %
**Men (n = 898)**					
R5†	present study	0.0323	10.61	0.0121	18.49
	Newbury	0.0965	31.69	0.0213	21.60
	Care Fusion	0.0007	0.23	0.0127	9.35
R20†	present study	0.0205	9.13	0.0058	12.81
	Newbury	0.0077	3.45	0.0059	5.90
	Care Fusion	-0.0396	-17.67	0.0076	3.34
X5^‡^	present study	-0.0159	14.36	0.0041	15.14
	Newbury	-0.0384	34.76	0.0056	24.94
	Care Fusion	-0.0900	81.41	0.0122	8.69
**Women (n = 913)**					
R5†	present study	0.0340	8.93	0.0143	15.99
	Newbury	0.0158	4.15	0.0151	5.26
	Care Fusion	-0.0200	-5.24	0.0156	5.15
R20†	present study	0.0167	6.12	0.0055	8.11
	Newbury	-0.0300	-11.00	0.0064	1.42
	Care Fusion	-0.0674	-24.72	0.0104	0.66
X5^‡^	present study	-0.0241	16.25	0.0056	18.62
	Newbury	-0.0148	9.99	0.0057	8.32
	Care Fusion	-0.0573	38.65	0.0091	7.01

† above upper limit of normal (ULN), ^‡^ below lower limit of normal (LLN).

## Discussion

Based on a random population sample from Germany of Caucasian origin, reference equations for all IOS indices were determined for adults particularly covering the advanced age range. Care was taken to include only lung healthy subjects with no history of smoking or lung disease and spirometric values above the LLN [24]. Subjects were fairly evenly distributed across the age range studied, with some emphasis on advanced age but thinning-out beyond 85 years. Based on the characteristics of our population we suggest that the reference equations derived for the median, ULN and LLN of IOS indices should be applied only in subjects aged between 45 to 85 years, with typical body weights (55 to 100 kg) and heights (160–190 cm in men and 145–170 cm in women). As previously reported [2], [14], [15], Rrs was higher in females than males, while the opposite was true for Xrs at low frequencies. Some of the IOS indices were age-dependent, but not as pronounced as spirometric indices [24], [29]. In line with physiological and morphological changes occurring in old age [18], [19] age-dependent changes were most prominent for X5, AX, Fres, and R5–R20 (Fig. S2). All of these are suggested to be associated with peripheral lung function [32], although other mechanisms such as an airway shunt proximal to an elevated resistance or inhomogeneous constriction of peripheral airways may also hold true.

In addition to the linear models we also tested more complex regression models, e.g. additive polynomial ones, with various functions of age, height, weight, or BMI. It turned out, however, that the linear models, with age, height and body weight as predictors of IOS indices, fitted best. This is in accordance with previous findings [15] although other models for IOS indices have been reported in Japanese population [17]. While body weight was a significant predictor for most IOS parameters in females, its relevance was less obvious in men, particularly for the 95% percentile. Respiratory mechanics is known to be affected by increased body weight [33], [34] resulting in decreased FRC, expiratory reserve volume and compliance, as well as abnormalities of ventilation even with a modest increase in weight. This raises the risk of expiratory flow limitation and airway closure [35]. Although our epidemiological setting does not provide evidence, particularly the latter may be involved in the functional link between body weight and various IOS indices in our study.

Predictive equations for IOS indices of adults are scarce. Only two studies, by Vogel & Smidt and Newbury et al. [2], [15] have been published for Caucasians and one study for Japanese [17]. There were marked differences between our and the published equations for Caucasians (Fig. 1), which were more pronounced in men than women when quantified by the differences between observed and predicted values (Table 5). The comparison of estimates in ficticious, average height and weight subjects (Fig. 2) underlined the differences in Rrs in both sexes, while values of Xrs appeared to be more comparable. Several reasons may account for these differences. Firstly, they could be related to the inclusion criteria of study populations. Our study was based on a randomly selected population sample, while Newbury et al. [15] used purposive sampling of healthy individuals which could have introduced a sampling bias. On the other hand, it provided equal numbers for both sexes, which we did not achieve as females outnumbered males by 50%. Smokers were included in the population by Vogel & Smidt [2], and Newbury et al. [15] included some past smokers, whereas we strictly excluded subjects with a smoking history. Moreover, the number of subjects varied between studies, the lowest being just above 100 [15]. Both published studies did not cover ages beyond 75 years, while we put emphasis on the middle and advanced age range. In all studies subjects were Caucasians but they originated from different countries, Australia [15] vs. Germany (Vogel & Smidt [2] and our study). As for spirometry, this could affect reference equations [24], [36], [37].

It is also important that different statistical approaches have been employed. The published equations were based on standard linear regression models that rely on data properties which may be violated by IOS data, especially regarding normal distribution and homoscedasticity. Indeed, the Shapiro-Wilk test showed that most of resistance and reactance indices were not normally distributed (Fig. S1). Therefore, we used quantile regression which is free of assumptions on the distribution of outcome measures and more robust against outliers or skewed distributions [28]. The comparison of prediction equations thus requires some caution, since different statistical parameters are involved, e.g. mean vs. median. For instance, in case of the right skewed distribution of resistance indices the mean value is somewhat higher than the median. The generalized additive models for location, scale and shape [29], which have been recently applied to spirometric indices could have been an alternative to quantile regression. However, quantile regression appeared to be better suited with respect to the large but limited number of subjects included in our monocentric study. It also better allows for the comparison and interpretation of results.

As a result of the possible explanations for differences in IOS predictive equations we would like to suggest that the present equations are the best to use in Caucasians, particularly for ages between 45 to 85 years. We also provide reference values for recently recognized IOS indices such as AX. Subjects of advanced age represent a population of great interest for IOS as the cooperation in spirometry is often limited. However, the clinical and predictive relevance of IOS in that age range remains to be investigated in clinical studies.

The potential clinical implications of the use of different reference equations were studied by applying them to the entire KORA study population (Table 6). The mean differences between observed values and predictions were considerably higher in both sexes for R20 and X5 when using the Vogel & Smidt equations. The percentages of subjects beyond the limits of normal were comparable between our and these predictions for R5 but much lower for R20 in both sexes, while small differences were found for X5 in woman. This demonstrates that with the reference equations currently applied by the IOS providing company evaluation of data results in systematic differences.

### Conclusions

Our study provides up-to-date reference equations for all common indices of IOS in Caucasians of age 45 to 85 years. To facilitate the detection of functional alterations by IOS in this age range, we suggest to the use of the novel prediction equations. Still, further studies from Caucasians of advanced age are required to confirm our results and provide a more sound basis of IOS reference equations for clinical and epidemiological use.

## Supporting Information

Figure S1 **Frequency dependency of resistance and reactance.** Resistance and reactance between 5 Hz and 35 Hz of the lung healthy study population is provided for men (n = 154) and women (n = 243). # indicate significant differences between men and women (p<0.05)
(TIF)

Figure S2 **Age dependency of IOS indices.** Individual values of men and women are provided for different IOS indices. Further, the median (solid), 5th (dotted) and 95th (dashed) percentile of a subject with median height and weight values of our study population is shown.
(TIF)

Table S1 **Reference equations of IOS indices for men and women.** Provided are quantile regression equations for the IOS indices R10, R15, R25, R35, and X10 to X35 for median, 5th and 95th percentile with age in years, height in cm and weight in kg.
(DOC)
