# Supplementary figures and images for: Reference Values of Impulse Oscillometric Lung Function Indices in Adults of Advanced Age
Source: PLoS One. 2013 May 15;8(5):e63366. doi: 10.1371/journal.pone.0063366 (PMC3655177; doi:10.1371/journal.pone.0063366)

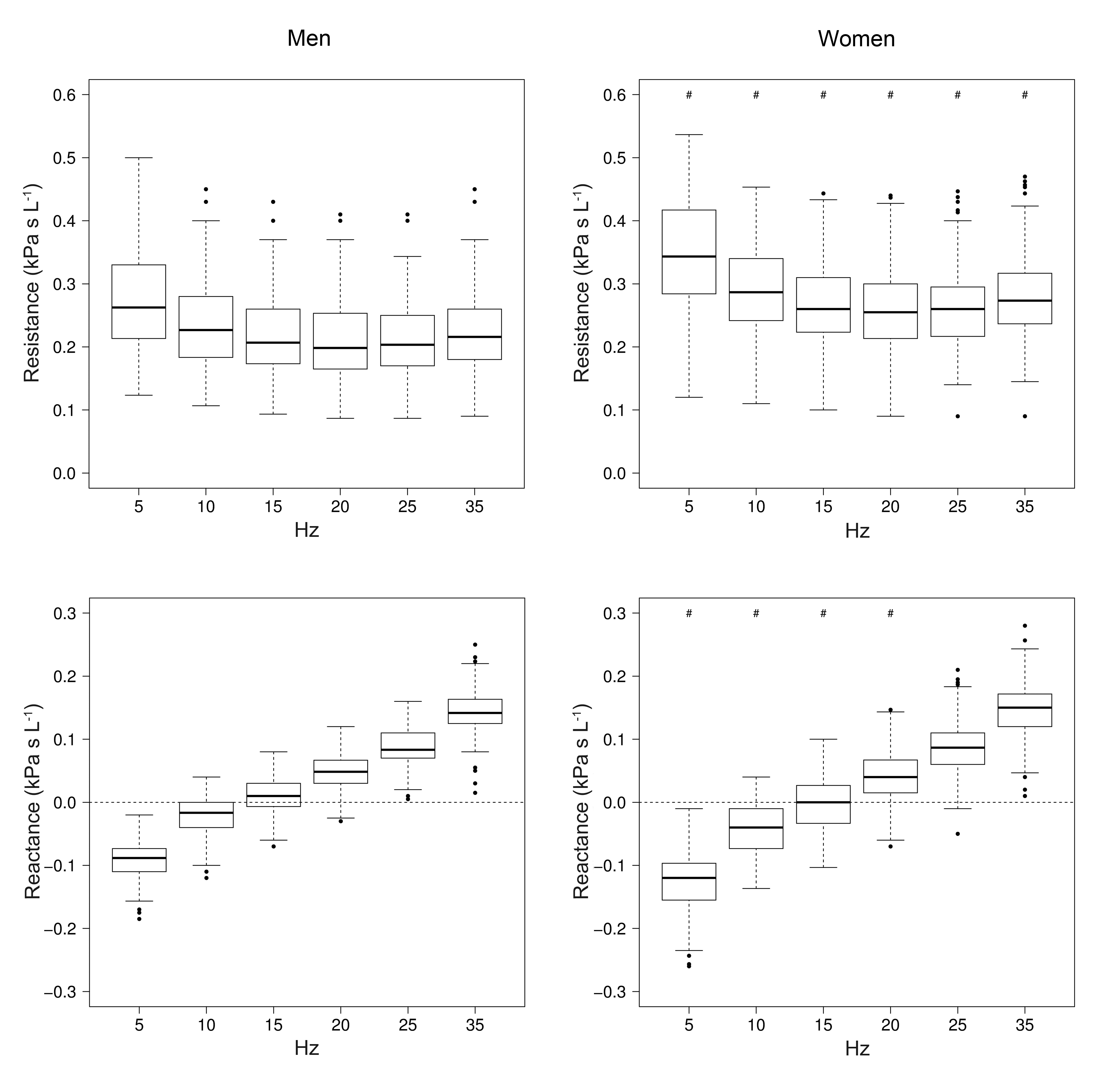

Supplement: Figure S1 — Frequency dependency of resistance and reactance. Resistance and reactance between 5 Hz and 35 Hz of the lung healthy study population is provided for men (n = 154) and women (n = 243). # indicate significant differences between men and women (p<0.05) (TIF) [file pone.0063366.s001.tif]

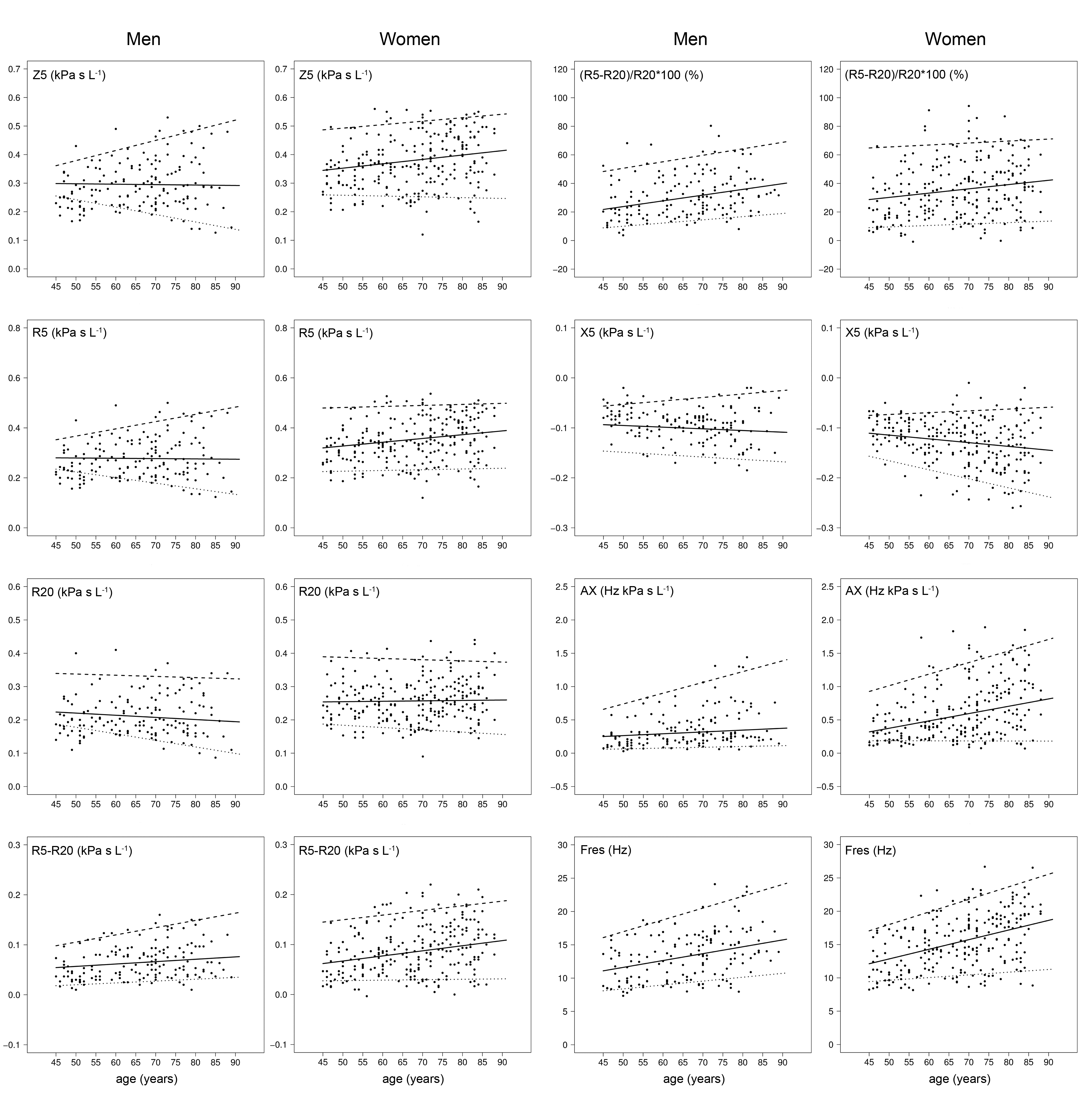

Supplement: Figure S2 — Age dependency of IOS indices. Individual values of men and women are provided for different IOS indices. Further, the median (solid), 5th (dotted) and 95th (dashed) percentile of a subject with median height and weight values of our study population is shown. (TIF) [file pone.0063366.s002.tif]
