# Supplementary material for: Reference Values of Impulse Oscillometric Lung Function Indices in Adults of Advanced Age
Source: PLoS One. 2013 May 15;8(5):e63366. doi: 10.1371/journal.pone.0063366 (PMC3655177; doi:10.1371/journal.pone.0063366)
Supplement: Table S1 — Reference equations of IOS indices for men and women. Provided are quantile regression equations for the IOS indices R10, R15, R25, R35, and X10 to X35 for median, 5th and 95th percentile with age in years, height in cm and weight in kg. (DOC) [file pone.0063366.s003.doc]

**Table S1: Reference equations of IOS indices for men and women**

| **Equations for men** | |  |  | **Coefficients of** | | |
| --- | --- | --- | --- | --- | --- | --- |
| **Percentile** | | **Quantile value** | **Intercept** | **Age** | **Height** | **Weight** |
| R10 (kPa s L^-1^) | 5% | 0.15 | 0.7608053 | -0.0018970 | -0.0028262 |  |
|  | 50% | 0.23 | 0.8500105 | -0.0002916 | -0.0044800 | 0.0021392 |
|  | 95% | 0.36 | 1.1499931 | 0.0005800 | -0.0048548 |  |
| R15 (kPa s L^-1^) | 5% | 0.14 | 0.4788628 | -0.0008444 | -0.0023545 | 0.0015407 |
|  | 50% | 0.21 | 0.8484545 | -0.0004936 | -0.0042916 | 0.0016870 |
|  | 95% | 0.33 | 1.2066933 | 0.0000320 | -0.0051061 |  |
| R25 (kPa s L^-1^) | 5% | 0.14 | 0.6088613 | -0.0016253 | -0.0020911 |  |
|  | 50% | 0.20 | 0.7134742 | -0.0008100 | -0.0033337 | 0.0014541 |
|  | 95% | 0.31 | 1.0242570 | -0.0002410 | -0.0040161 |  |
| R35 (kPa s L^-1^) | 5% | 0.15 | 0.6741496 | -0.0019355 | -0.0023167 |  |
|  | 50% | 0.22 | 0.7084185 | -0.0008388 | -0.0030919 | 0.0012065 |
|  | 95% | 0.34 | 1.0792848 | -0.0005261 | -0.0041102 |  |
| X10 (kPa s L^-1^) | 5% | -0.08 | -0.0737045 | -0.0016742 | 0.0006061 |  |
|  | 50% | -0.02 | -0.1340850 | -0.0005945 | 0.0014990 | -0.0012642 |
|  | 95% | 0.02 | -0.0930851 | -0.0005851 | 0.0008511 |  |
| X15 (kPa s L^-1^) | 5% | -0.03 | -0.2588542 | -0.0009606 | 0.0016204 |  |
|  | 50% | 0.01 | -0.1034239 | -0.0007079 | 0.0014861 | -0.0011579 |
|  | 95% | 0.05 | -0.0579056 | -0.0008260 | 0.0013864 | -0.0009735 |
| X20 (kPa s L^-1^) | 5% | 0.01 | 0.0971653 | -0.0010465 | -0.0001072 |  |
|  | 50% | 0.05 | 0.0255885 | -0.0009716 | 0.0009511 | -0.0009383 |
|  | 95% | 0.09 | -0.1177028 | -0.0008434 | 0.0021006 | -0.0012936 |
| X25 (kPa s L^-1^) | 5% | 0.03 | 0.4318730 | -0.0019617 | -0.0015055 |  |
|  | 50% | 0.08 | 0.1563108 | -0.0010368 | 0.0004089 | -0.0008866 |
|  | 95% | 0.13 | 0.1157309 | -0.0008193 | 0.0004159 |  |
| X35 (kPa s L^-1^) | 5% | 0.09 | 0.7404901 | -0.0028461 | -0.0027085 |  |
|  | 50% | 0.14 | 0.2938889 | -0.0008333 | -0.0005556 |  |
|  | 95% | 0.20 | 0.3264791 | -0.0008062 | -0.0004000 |  |

| **Equations for women** | |  |  | **Coefficients of** | | |
| --- | --- | --- | --- | --- | --- | --- |
| **Percentile** | | **Quantile value** | **Intercept** | **Age** | **Height** | **Weight** |
| R10 (kPa s L^-1^) | 5% | 0.18 | 0.4809424 | 0.0002429 | -0.0033526 | 0.0033552 |
|  | 50% | 0.29 | 0.6897185 | 0.0009021 | -0.0039559 | 0.0024615 |
|  | 95% | 0.42 | 0.4719983 | 0.0005201 | -0.0005654 |  |
| R15 (kPa s L^-1^) | 5% | 0.17 | 0.5231851 | -0.0003587 | -0.0031551 | 0.0026231 |
|  | 50% | 0.26 | 0.5258876 | 0.0003329 | -0.0026050 | 0.0019214 |
|  | 95% | 0.38 | 0.2962761 | 0.0013976 | -0.0000284 |  |
| R25 (kPa s L^-1^) | 5% | 0.17 | 0.5883734 | -0.0006121 | -0.0032050 | 0.0020710 |
|  | 50% | 0.26 | 0.3118373 | 0.0002711 | -0.0010112 | 0.0012832 |
|  | 95% | 0.38 | 0.5796914 | 0.0002160 | -0.0013580 |  |
| R35 (kPa s L^-1^) | 5% | 0.19 | 0.4405811 | -0.0011068 | -0.0011100 |  |
|  | 50% | 0.27 | 0.5139098 | -0.0002254 | -0.0019556 | 0.0012826 |
|  | 95% | 0.40 | 0.3717460 | -0.0007823 | 0.0005140 |  |
| X10 (kPa s L^-1^) | 5% | -0.12 | -0.3144773 | -0.0008281 | 0.0023341 | -0.0015115 |
|  | 50% | -0.04 | -0.1933200 | -0.0010049 | 0.0019482 | -0.0013264 |
|  | 95% | 0.01 | -0.0959890 | -0.0006708 | 0.0014556 | -0.0012597 |
| X15 (kPa s L^-1^) | 5% | -0.07 | -0.0342091 | -0.0014215 | 0.0007784 | -0.0007871 |
|  | 50% | 0.00 | -0.1428449 | -0.0012609 | 0.0019050 | -0.0011204 |
|  | 95% | 0.06 | -0.1558996 | -0.0007236 | 0.0022713 | -0.0015288 |
| X20 (kPa s L^-1^) | 5% | -0.02 | -0.0168431 | -0.0015098 | 0.0006863 |  |
|  | 50% | 0.04 | -0.0114201 | -0.0013149 | 0.0012569 | -0.0008589 |
|  | 95% | 0.11 | 0.0452323 | -0.0013353 | 0.0016874 | -0.0017546 |
| X25 (kPa s L^-1^) | 5% | 0.02 | -0.0444164 | -0.0014320 | 0.0010830 |  |
|  | 50% | 0.09 | 0.0510047 | -0.0014803 | 0.0010831 | -0.0005790 |
|  | 95% | 0.16 | 0.0748510 | -0.0016454 | 0.0011557 |  |
| X35 (kPa s L^-1^) | 5% | 0.08 | 0.1406846 | -0.0014576 | 0.0002530 |  |
|  | 50% | 0.15 | 0.2002944 | -0.0013822 | 0.0002495 |  |
|  | 95% | 0.22 | 0.2496872 | -0.0016145 | 0.0004201 |  |

Provided are quantile regression equations for the IOS indices R10, R15, R25, R35, and X10 to X35 for median, 5th and 95th percentile with age in years, height in cm and weight in kg. (e.g., for women: R10-50% = 0.6897185 + 0.0009021 age -0.0039559 height + 0.0024615 weight)
